# Implementing a Mental Health Care Program and Home-Based Training for Mothers of Children With Autism Spectrum Disorder in an Urban Population in Bangladesh: Protocol for a Feasibility Assessment Study

**DOI:** 10.2196/resprot.8260

**Published:** 2017-12-14

**Authors:** Aliya Naheed, Kamrun Nahar Koly, Helal Uddin Ahmed, Shaheen Akhter, MM Jalal Uddin, Mary C Smith Fawzi, Subhash Chandir, Muzharul Mannan, Saima Hossain, Charles Nelson, Kerim Munir

**Affiliations:** ^1^ Initiative for Non-Communicable Diseases Health Systems and Population Studies Division International Centre for Diarrhoeal Disease Research, Bangladesh Dhaka Bangladesh; ^2^ National Institute of Mental Health, Bangladesh Dhaka Bangladesh; ^3^ Institute for Paediatric Neurodisorder & Autism Bangabandhu Sheikh Mujib Medical University Dhaka Bangladesh; ^4^ National Institute of Neurosciences & Hospital Dhaka Bangladesh; ^5^ Global Health and Social Medicine Harvard Medical School Cambridge, MA United States; ^6^ Center for Global Health Delivery-Dubai Harvard Medical School Dubai United Arab Emirates; ^7^ Shuchona Foundation Dhaka Bangladesh; ^8^ Laboratories of Cognitive Neuroscience Boston Children's Hospital Boston, MA United States; ^9^ Division of Developmental Medicine Boston Children’s Hospital Boston, MA United States

**Keywords:** depression, psychosocial, counseling, autism spectrum disorder, mothers, training

## Abstract

**Background:**

Mothers of children with autism spectrum disorder (ASD) have reported a higher level of depression than mothers of children with other neurodevelopmental disorders in both developed and developing countries. Mothers are the lifetime caregivers of children with ASD, and a high burden of depression can negatively impact their ability to provide care. However, access to mental health services in primary care is limited, given the scarcity of qualified providers in Bangladesh.

**Objective:**

We aim to pilot the feasibility of integrating mental health services for the mothers of children with ASD attending schools offering ASD care and improve skills of mothers for child care through a home-based training program.

**Methods:**

The study will be conducted in two selected schools in Dhaka in Bangladesh that have been offering services for ASD for more than 10 years. A female psychologist will be deployed at the schools to offer nonpharmacological services for all mothers having a depressive episode. Referral for pharmacological treatment will be made at the discretion of supervising psychiatrists. An ASD special educator will provide training to the mothers for enhancing their child care skills at home on a monthly basis. The proposed intervention package will be implemented over a period of 4-6 months, and the feasibility of the intervention will be assessed through a pre- and postintervention evaluation by obtaining the perspectives of various stakeholders involved in the implementation of mental health services and maternal training. The primary outcome will include assessment of acceptability, adaptability, demand, practicality, implementation, and integration of the package intervention in the school settings. The secondary outcomes will include assessment of: 1) the prevalence of maternal depression; 2) children’s behavioral, social, and communication skills; and 3) the intervention participation costs incurred by institutions and families.

**Results:**

Between February and March 2017, 188 mothers of children with ASD were screened for depression following a written informed consent. Based on the Diagnostic and Statistical Manual of Mental Disorders, 4th edition (DSM-IV), the Structured Clinical Interview for the DSM-IV (SCID-1) was administered to 66 mothers. In-depth interviews were conducted with 10 mothers and 8 various stakeholders. Between January-June 2017, the team finalized a draft psychosocial counseling module and a maternal training module. Between April-May 2017, mental health services were provided by psychologists to 41 mothers who attended the counseling centers at each school. Three special educators have been trained in June 2017 to initiate training of the participating mothers.

**Conclusions:**

This is the first study of a mental health intervention for mothers of children with ASD to reduce their burden of depression and improve the outcomes of their children. The findings will inform the provision of services for children with ASD and their mothers in Bangladesh and similar settings.

## Introduction

### Background

The global burden of depression accounts for 2.5% of the Disability Adjusted Life Years (DALYs). In the South Asia region, the proportion of burden due to depression is 5-times higher at 13.3% of DALYs per 100,000 populations [1]. The prevalence of maternal depression in the South Asian countries, ranging between 23% and 32% [2-4], is also higher than the estimate in other low- and middle-income countries (15.6 %) [5]. There is a dearth of information on the specific burden of depression in individual South Asian countries, including Bangladesh [6].

The prevalence of autism spectrum disorder (ASD), a neurodevelopmental disorder, has increased exponentially worldwide, including Bangladesh [7-9]. The global burden of ASD accounts for 53 DALYs per 100,000 population [7]. A review of ASD prevalence in Asian countries suggests a range between 1.9 and 14.8 per 10,000 [10]. Mothers of children with ASD have reported higher level of depression than mothers of children with other neurodevelopmental disorders in both developed and developing countries [11], for example, 48% versus 38% in Pakistan and 8% versus 4% in Sweden. Research has documented that parenting a child with ASD can have significant negative impact on the parents’ quality of life [12,13]. In Bangladesh, according to a national survey conducted in 2013, the prevalence of ASD was 15.5 per 10,000 children, and higher prevalence was reported in urban areas (300 per 10,000 children) than the rural areas (6.8 per 10,000 children), although the report did not provide an explanation of this regional variation [14]. To our knowledge, there has been little scholarly research published on the burden of maternal depression among children with ASD in Bangladesh.

In 2015, Naheed and colleagues at the International Centre for Diarrhoeal Disease Research, Bangladesh (icddr,b) estimated that 45% of the mothers of ASD children and adolescents aged 3-17 years, who were enrolled in a special school for ASD, had suffered an episode of major depressive disorder (MDD), half of whom reported a current major depressive episode (MDE) [15,16]. Remarkably, most mothers (70%) had a college degree or a higher level of education [14,15].

The high impact of depression among mothers of children and adolescents with ASD therefore underscored the dire need for provision of better quality of mental health care to support them in caring for their children with ASD. Mothers are the lifetime caregivers of children with ASD, and a high burden of depression is likely to negatively impact their ability to care for their children at home [17,18]. Regrettably, like many other countries in South Asia, there is a scarcity of human resources in Bangladesh to provide mental health services. Furthermore, within the mental health departments at the teaching hospitals and specialty hospitals such as the National Mental Health Institute, Bangladesh, many mothers of children with ASD do not prefer receiving care at a mental health facility due to their fear of stigmatization coupled with lack of anticipated support for them in the hospital environment (*personal communication with Aliya Naheed, June 2015*). Indeed, the ASD care programs in Bangladesh are largely limited to institutions based mostly in urban areas [15,19]. These services often are not geared to address the needs of the mothers in caring for their children at home and do not provide any long-term rehabilitation guidance for families.

An important shift for development of future ASD services has been recommended at a Bangladesh-sponsored Global Panel on ASD during the World Autism Day on April 2, 2016, held at the United Nations. There is an impetus in the country to address ASD as a major public health problem [20]. There is a strong political commitment of the Prime Minister of Bangladesh for creating a long-term rehabilitation opportunity for children with ASD and other neurodevelopmental disabilities (as interlinked by the World Health Assembly) under the stewardship of the government [21]. The Bangladesh Government has been taking steps in recent years to develop programs to meet the needs of ASD in the community, including capacity development of parents in ASD care [22]. Nonetheless, to date, the proposed programs have been heavily institution-focused services that primarily target ASD children. Training programs targeting parents have been limited in few schools in select areas. They have involved customized sessions on awareness building for mothers to better inform about the children’s needs, behaviors, and activities for daily living. They have not targeted comprehensive programmatic and training needs of the mothers to improve their ability to care for their children with ASD at home (*personal communication with Aliya Naheed, June 2015*). Furthermore, these parent-oriented programs have not been integrated with mental health services [20,23,24].

The parent-focused services for ASD require a high number of well-qualified educational psychologists with special training in ASD and neurodevelopment disorders (special educators), which is scarce in Bangladesh. Such parent-focused programs cannot be delivered by the community health workers who traditionally support basic maternal and child health services [25]. The deployment of such a trained workforce of special educators requires substantial resources, and the approach is likely to be time-consuming and expensive. An alternative proposal is to bring the mothers of children with ASD under a structured formal training program within specialized schools to which their access can be facilitated. These umbrella programs can enhance maternal skills for child care at home through organized training deploying a more limited cohort of special educators at schools. Furthermore, these educators can engage mothers to develop enhanced home-based services, thus leading to improved child performance and greater satisfaction by mothers at home [24]. Since maternal depression may be attributable to multitude of factors other than ASD in their children, introducing mental health services in the schools where mothers have access to institutional supports for their special needs children, is also likely to create a conducive environment for the mothers to avail mental health services. Creation of a mental health support system combined with the capacity building of mothers for supporting the care needs of their children has not been previously attempted. Furthermore, integrating mental health services in school-based program targeting ASD is an innovative approach and can provide wider outreach for mothers of children with ASD in the community in a low-resource mental health setting.

Cost is one of the key barriers to adopting new interventions in the existing programs in low- and middle-income countries, and cost parameters are often estimated through recall [26-28]. The proposed research will capture the direct cost of the intervention based on actual activities involving the intervention in real time and estimate all possible realistic costs that would be incurred by both the schools (provider cost) and the participating families (out of pocket cost) due to integrating the proposed combined intervention. Due to infeasibility of estimating indirect cost to families related to productivity loss, only the real-time direct cost estimates will be used to help researchers, policy makers, and institutions to design a large-scale trial or scale-up this intervention in the future. A further goal of the research is to assess the barriers to integrating the pilot intervention package with other types of facility-based services as well as prospect for scaling up of the intervention in other institutional settings in urban Bangladesh.

### Objectives

The primary objective of the study is to assess the feasibility of the proposed intervention at special schools for children with ASD that would provide mental health services and training to mothers suffering from a concurrent MDE. The secondary aim of the project is to assess the change in the prevalence of MDE among mothers and any improvement in the degree of individual performance of the children with ASD. Additional project aims include assessment of incremental direct institutional costs incurred at schools and out of pocket direct costs incurred by the families following the introduction of mental health services in combination with the maternal training program in the schools.

## Methods

### Design

This is a mixed-method feasibility study with a pre-post design.

### Study Setting

There are about 40 special schools providing variety of services targeting children with ASD and other neurodevelopmental disorders in Bangladesh. The majority of the schools are located in Dhaka centrally with 9 suburban schools. The study will be conducted in two selected schools in Dhaka city that have been providing high quality services for ASD over 10 years. On average, about 100 to150 children with ASD are registered in each school, and services offered at the schools for children with ASD are more or less similar in nature.

### Eligibility Criteria

The mothers of children with ASD, aged 18 years and older, with a child aged 3 to 17 years, registered in one of the two study schools, and given written voluntary consent will be recruited in the study. The mothers who are being treated for a severe behavioral or medical comorbid condition or who themselves are mentally or cognitively compromised or too ill to commute will be excluded from the study.

### Intervention Phase

The intervention package is planned with the following two components: (1) mental health care services at schools targeting the mothers diagnosed as having depression that have consented to participate in the study and (2) organized training sessions for supporting enhanced child care at home.

#### Implementing Mental Health Care Services at School

We will set up a counseling center at each school by deploying one trained female psychologist who would provide counseling to the intervention mothers. Currently, there is no structured counseling module available at the National Institute of Mental Health, Bangladesh (NIMH,B) or other institutions in Bangladesh. We will compile the documents available at NIMH,B and develop a counseling module with the help of an Expert Working Group (EWG) represented by psychiatrists, psychologists, and other relevant experts in Bangladesh (Table 1).

**Table 1:** Content of the psychosocial management module.

Module	Content
Psychoeducation	Psychoeducation refers to the education offered to individuals with a mental health condition and their families to help empower them and deal with their condition in an optimal way. Psychoeducation will be given by trained psychologists under the guidance of a senior psychologist to increase the self-esteem of the mothers, which will include the brief concept on depression, the probable etiology, how to mitigate the symptoms, compliance to the therapeutic process, and the consequences
Assessment of the strength and weakness of the mothers	The psychologists will assess the strengths and weaknesses of the mothers and discover their personal obstacles, issues that might hinder their progress in terms of social context, family support, financial state, educational qualification, and empowerment
Sharing the management plan with the mothers	The psychologists will share the whole management processes with the mothers, and any opinion from the mother could be a part of the management. The goal is to empower mothers and include them as a part of the management team counseling other mothers
Reconstruction of the cognition	Cognitive restructuring is a psychotherapeutic process of learning to identify and dispute irrational or maladaptive thoughts known as cognitive distortions. It is a core part of cognitive behavioral therapy (CBT). CBT is one of the most effective psychological treatments for common problems like depression, anxiety disorders, and so on. The process involved in CBT identifies the cognitive distortion, tracks the accuracy of thinking process, tests the thoughts, evaluates the evidence against the distorted thoughts, and assesses mindfulness and self-compassions, which are the core features of cognitive restructuring
Behavior therapy-graded activity	Graded activity is a principle of therapeutic intervention in which tasks are classified and gradually presented according to the client’s level of function and the challenge or degree of skill (physical, social, or cognitive) required by the task. The graded activity starts from personal care, daily household activities, child care, engagement in small and easy tasks, and complex and productive tasks. In this study, after assessing the strengths and weaknesses of the mothers, we will monitor and try to increase their graded activity.
Developing a mother’s community and engaging mothers in community-related activities	The psychologist will arrange a workshop in each participating school at 2-month intervals for enabling mothers to meet other mothers in the intervention program and create a common platform where they can share their experiences and success stories and encourage a positive reinforcement from depressed mothers. The mothers will be encouraged to participate in the social awareness or other activities arranged by the schools so that the mothers refrain from social isolation and rebuild their self-esteem

A psychologist will decide on the number of sessions and intervals between consecutive sessions based on the need of a mother. All eligible participants would provide voluntary consent (Multimedia Appendix 1). Before every session the psychologist will assess the mental health state by applying Beck Depression Inventory (BDI), which has been validated in Bangladesh [29]. Every month, a psychiatrist from NIMH,B will visit each school and review the records maintained by the psychologist and provide necessary advice regarding the need for any additional care of the mothers identified with MDE. The psychiatrist will reassess the additional need of a mother with MDE following face-to-face consultation if suggested by the record review or clinically identified by the psychologist. The medical care will therefore focus on management of depression with varying degrees of severity. The care will follow the management guidelines including referral to the National Institute of Mental Health for advanced mental health care if necessary (Table 2). All mothers referred to the NIMH,B for providing advanced mental health care will be eligible to receive free antidepressant, anxiolytic, and antipsychotic medications as deemed clinically appropriate.

#### Organized Training for Mothers With MDE in the Schools for Supporting Child Care at Home

One special educator will be deployed at each school who will organize structured training sessions for the intervention group of mothers following a training module developed by icddr,b in 2015 titled “Bangladesh Parent Empowerment Program (BPEP)” (*personal communication with Aliya Naheed, June 2015*). The training module will be customized according to the local context under the guidance of a group of experts on ASD. The BPEP module was piloted with 56 parents and validated for its *application* in the local setting (*personal communication with Aliya Naheed, June 2015*). Before training, the special educator will interview the mothers for validating children’s ASD diagnosis and rate the ASD. The educator will also interview the mothers to assess the individual performance of their children by using a standard ASD Diagnostic Check-List (ADCL) [30]. The ADCL will provide an assessment on individual performance of the ASD children of the mothers with MDE enrolled in the intervention activities (Multimedia Appendix 2, study activity flowchart). ADCL is a standard tool applied by ASD experts in Bangladesh to track improvement in performance in children with ASD. This tool comprises 60 items focusing on 6 major domains: (1) General Observation (13 items); (2) Cognition (10 items); (3) Emotion (8 items); (4) Social/Self-Help (8 items); (5) Communication (12 items); and (6) Sensory deficiency (9 items). ADCL will be applied to assess the degree of ASD in children at baseline and also in end-line to assess any potential change in the performance of the children following the BPEP training.

**Table 2:** Participant timeline.

Activities	Prestudy	Q^a^1	Q2	Q3	Q4	Q5
Institutional Review Board	X^b^					
Customization of the training module and development of the training materials		X				
Development of the psychosocial counseling module		X				
Setting up the counseling centers		X				
Staff training		X				
Recruitment of study participants		X				
Preintervention survey at two schools		X				
Preintervention feasibility interviews		X				
Provide mental health services at schools			X	X		
Home-based maternal training by special educators at the schools			X	X		
Monthly home-based refreshers training			X	X		
Postintervention survey at two schools					X	
Postintervention feasibility interviews						
Data entry and management		X	X	X	X	
Data analysis and report writing						X
Dissemination						X
Final project report submission						X

^a^Q: one-quarter of a year (3-month period).

^b^X: accomplishment of an activity.

The special educator will conduct multiple group sessions (with 5-8 mothers in each group) for covering the nine modules over a 4-week timetable following the module that will be developed by the local experts (Table 3). The special educators will follow-up with mothers at home every month for conducting refresher training and document the need for additional training supports for a child. The special educator will consult the icddr,b coinvestigators about the additional training need of a child and incorporate necessary training as advised, if required.

### Outcome

The primary outcome of the study is to determine the feasibility of integrating mental health care services at special schools for the mothers suffering from current MDE combined with the maternal training program for supporting child care at home [31]. The facilitators and barriers of the multi-component intervention at different levels will be assessed by conducting focus group discussions with the recipients and those who were involved in the delivery of the services and evaluated through pre-post qualitative interviews to assess the outcome parameters following the standard feasibility guideline [32,33]. The indicators will include acceptability, demand, implementation, practicality, integration, and adaptation (Table 4). The following aspects will also be assessed through in-depth interview (IDI) and key informant interview (KII) at various stakeholders’ levels:

- Knowledge, attitude, and practice about depression and management of depression for mothers of children with ASD
- Barriers for mothers for accessing institutional care for mental health and training at home (logistic, cost, family supports, time, etc)
- Community supports required for complying with the intervention
- The barriers of setting up intervention at an institution (administrative, logistics, cost, policy, etc)
- Additional supports that would be required for implementing mental health services at schools for ASD and for continuing the training program at home

The secondary outcomes of the study will assess the potential of the combined interventions’ impact based on the following measurable parameters as listed below through pre- and postintervention surveys:

1. Change in the prevalence of MDE among the mothers of children with ASD enrolled in the two selected schools: assessment of MDE in mothers of all children who have been recruited in the study before and after the intervention to evaluate change on the burden of MDE due to the combined intervention. This assessment will be done irrespective of the presence of depression status or other mental health conditions at the baseline to assess any spillover effect of the intervention on mothers in general.
2. Change in the degree of individual behavioral, social, and communication skills of ASD children whose mothers have received the intervention: the goal is to assess if the mental health service that mothers would have access to might influence any positive change in child’s behavior, communications, and social skills irrespective of providing additional training to mothers for child care at home. Individual performance of the children will be assessed before and after intervention to assess change in individual performance of their children following the combined intervention.
3. Incremental costs incurred to the selected schools following introduction of mental health services in combination with the maternal training program: the project will estimate the overall direct costs that would be incurred by an individual institution due to the introduction of the combined services at the school. As such, cost of mental health services will be tracked throughout the intervention period of 6 months which will include counseling sessions, psychiatrists’ visits, cost of services obtained at the mental health care facility, or any other costs that might be potentially borne by the institutions for providing mental health care at their schools. The cost of maternal training program will include costs of conducting training of mothers at school over a 4-month period, cost for training sessions, salary of the special educators, training materials for mothers, and any other cost that might be potentially borne by the institutions for supporting this training.
4. Incremental costs incurred to the families (out of pocket cost) for supporting mental health services for participating mothers and child care at home following maternal training: the goal is to assess the additional direct costs incurred by a family to support mental health services for a depressed mother and costs incurred in order to enhance the child’s overall performance following adoption of the proposed training program for mothers. As such, we will track out of pocket costs of mental health services throughout the intervention period of 6 months and cost of conducting training of mothers at the school over a period of 4 months. Out of pocket costs borne by the study participants will include travel to health care facility or specialized institution for ASD care, medicines for treating both mother and child, teaching aids for supporting maternal training, fee for visiting an autism expert or other providers, recreational activity of the child with ASD, and any other costs relevant to the intervention that may be incurred by the mother during the intervention period.

Any potential positive change in MDE or child performance would indicate acceptability of the programs by both the stakeholders for its adaption in to the existing school services. Such changes would also indicate demand for the services, practicality for its implementation in the current setting, and potential for integrating in to a broader program for rendering a sustainable service to the target community. In case the proposed comprehensive and multilevel mental health service for addressing depression among mothers of children with ASD is feasible but not affordable by either the schools or families due to a high incremental costs, it would indicate that the proposed intervention would not be feasible for its integration in the existing services of ASD. The threshold for affordability will be determined in consultation with the EWG.

### Sample Size

This is a feasibility study; therefore, statistical power calculation was not carried out. The two selected schools would have at least 100-150 children with ASD registered in a given year. On the basis of the previous study findings, we anticipate that about 26% mothers would be diagnosed as having MDE, and we would find a total of 52 to 78 mother-child pairs from two schools for study recruitment [15].

For the qualitative surveys, we will conduct 15-20 key informant interviews including two respondents from each of the following categories: psychologists, psychiatrists, managers at schools, mothers without depression, family members of the depressed mothers, principals, special educators and relevant policy makers, and pediatric neurologists to assess the feasibility of the intervention.

### Recruitment

All eligible mothers will be contacted over phone with the permission of the school authorities and sent invitation to participate in the study. The invitation letters will briefly describe depression and its complications in general terms and the importance of early management. The letter will also provide information about the nature of the intervention, the evaluation process, and the purpose of the study in improving ASD care in schools and at home. The parents who would respond positively will be invited to attend an orientation session for discussing the project in detail and will get an opportunity to ask questions for any clarification. A group of trained research staff will individually contact each eligible mother either at home or a convenient location and recruit in the study following a written informed voluntary consent (Multimedia Appendix 1).

The trained research staff will apply a tool (Patient Health Questionnaire, PHQ-9) to screen depression in all recruited mothers based on nine criteria on DSM-IV (*Diagnostic and Statistical Manual of Mental Disorder*, 4th edition) using a Likert scale within a possible range of 0-27 (0=not at all, 1=several days, 2=more than half the days, and 3=nearly every day) [34,35]. On the basis of PHQ-9, the levels of depression on screening will be defined as None=0, Minimal=1-4, Mild=5-9, Moderate=10-14, Moderately Severe=15-19, and Severe=20-27. Those who will be defined as having a level of depression more than minimal (score >4) will be further assessed by a trained psychologist using the standard diagnostic tool (SCID-I Research Version) for confirmatory diagnosis of depression [36]. Mothers who would have MDE will be recruited in the intervention following additional consent (Multimedia Appendix 1). Additionally, we will select various stakeholders for conducting key informant interviews following a written informed voluntary consent (Multimedia Appendix 1).

**Table 3:** Summary of the content of the BPEP (Bangladesh Parent Empowerment Program) training.

Number and name of the module		Components included	Topics covered	Process of delivery
**Session 1**				
	1. Understanding autism	PowerPoint slides Video Feedback and experience sharing	What is autism? Characteristics of autism Prevalence, diagnosis, treatment, cause, and risks of autism Developmental milestone of ASD^a^ and normal child Myth and impact of autism Guidance and homework for mothers	Group session (5-6 mothers) (60 min) Practical demonstration Role play
	2. Communication	PowerPoint slides Uses of flash cards Individual activities Feedback and experience sharing	What is communication? Communication ability of ASD and normal children Techniques to develop communication ability of ASD children Techniques to increase eye contact and picture exchange communication system Guidance and homework for mothers	Group session (5-6 mothers) (60 min) Practical demonstration Role play
**Session 2**				
	3. Teaching your child language	PowerPoint slides Uses of flash cards with real objects Feedback and experience sharing	Language developmental stage of ASD children Techniques to develop language stage Guidance and homework for mothers	Group session (5-6 mothers) (60 min) Practical demonstration Role play
	4. Social communication	PowerPoint slides Feedback Checklist of social communication Uses of flash card Feedback and experience sharing	What is social communication? Social communication problems of ASD children Techniques to develop social communication ability Imitation and playing techniques for ASD children Guidance and homework for mothers	Group session (5-6 mothers) (60 min) Practical demonstration Role play
**Session 3**				
	5. Regulating your child’s behavior	PowerPoint slides Group discussion Feedback and experience sharing	Behavior of ASD children Techniques to manage ASD child’s behavior Guidance and homework for mothers	Group session (5-6 mothers) (60 min) Practical demonstration Role play
	6. Setting up your home for success routine and structure	PowerPoint slides Uses of flash card on home routine Feedback and experience sharing	Preparing work environment for ASD children Techniques to select toys and play with ASD children Techniques to teach to follow instruction Increasing child’s participation toward home activities Guidance and homework for mothers	Group session (5-6 mothers) (60 min) Practical demonstration Role play
**Session 4**				
	7. Community living	PowerPoint slides Feedback and experience sharing	Society living techniques for ASD children Guidance and homework for mothers	Group session (5-6 mothers) (60 min) Practical demonstration Role play
**Session 5**				
	8. Sensory regulation	PowerPoint slides Group discussion Feedback and experience sharing	Sensory difficulties of ASD children Techniques to reduce sensory difficulties Guidance and homework for mothers	Group session (5-6 mothers) (60 min) Practical demonstration Role play
	9. Food and nutrition	PowerPoint slides Group discussion Feedback and experience sharing	Food and diet chart for ASD children	Group session (5-6 mothers) (60 min) Practical demonstration Role play

^a^ASD: autism spectrum disorder.

**Table 4:** Feasibility assessment (qualitative).

Indicator	Target	Method	Outcome
Acceptability	Mother School managers/administrators Psychologist Special educators Psychiatrist	IDI^a^ KII^b^	Satisfaction Intent to continue to avail services Perceived appropriateness of the intervention Fit within the existing goal and culture of the special schools Perceived positive or negative effects on the mothers, providers, and institutions
Demand	Mother School manager/administrators Psychologist Special educators Psychiatrist	IDI KII	Perceived need of the interventions Intention to use the services
Implementation	Mother School manager/administrators Special educators Psychologist Psychiatrist	IDI KII	Process of execution/evaluation Actual usage by the recipients Barriers and opportunities during implementation Amount and type of resources needed to implement the services from every stakeholders’ perspective (mothers, providers and school managers, etc) Success of the implementation
Practicality	Mother School manager/administrators Special educators Psychologist Psychiatrist	IDI KII	Efficiency (speed and quality) of the programs Positive/negative effects on participants and also on the institutions Ability to carry out every activity of the intervention by every stakeholder Cost analysis of the overall program
Adaptation	Psychologist School manager/administrators	KII	Degree to which similar outcomes are obtained in a new context Modifications that are required to accommodate programs in the new context
Integration	School manager Policy makers	KII	Perceived fit with the current infrastructure Perceived barriers and opportunities to integrate the program Perceived sustainability Scope and challenges to scale up Cost to the organization and policy bodies Fit with organization goal and culture Effects (positive/negative) on organization

^a^IDI: in-depth interview.

^b^KII: key informant interview.

### Data Collection

#### Preintervention Survey (Baseline)

A group of trained research staff will conduct preintervention surveys with all eligible mothers at two schools to obtain information on sociodemographic characteristics, child information, school information, and other relevant data. Quality of life will be assessed by using Euro-Qol five dimensions questionnaire (EQ-5D) [7].

All the above tools have already been validated in Bangladesh by previous icddr,b researches (Naheed et al, 2016, unpublished data). A mother identified with any MDE with suicidal ideation, intent or plan, or a prior history of suicide attempt will urgently be referred for hospital-based mental health services in the community and will not be included in the study (standard of care in Bangladesh).

Additionally, we will identify various stakeholders to conduct key informant interviews following a qualitative guideline We will select stakeholders involved in the following categories: psychologists, psychiatrists, the family members of the depressed mothers, school administrators, management at the referral hospitals, special educators, and pediatric neurologists to assess the feasibility of the intervention, as well as a randomly selected group of mothers without any depression (PHQ-9 score, 0-4). The trained qualitative researchers will conduct the interviews following a written consent. All the interviews will be digitally audio-recorded and transcribed. The interviews will be reviewed by an independent qualitative researcher at various stage of data collection to check validity and reliability of the data for further verification of the missing data.

#### Postintervention Survey (End Line)

After completion of the intervention, an end-line qualitative survey will be carried out in a subset of the study participants and various stakeholders to assess the indicators of the feasibility of the study. This will include 2-3 interviews with each of the following categories: psychologists, psychiatrists, managers at schools, mothers without depression, family members of the depressed mothers, principals, special educators and relevant policy makers, and pediatric neurologists. The end-line qualitative survey will assess views of various stakeholders about the directions for institutionalizing the proposed intervention based on their experiences in the intervention, particularly the barriers they have encountered during the intervention period.

### Data Management and Quality Assurance

This research protocol includes Data Safety Monitoring Plan (DSMP). The purpose of the DSMP is to provide a framework for appropriate oversight and monitoring of the data collection and management to ensure the privacy of the participants and the validity and integrity of the data. The data collection tools will be pretested and checked for validity and reliability before going for data collection. All the study staff will be trained to promote standardized and objective collection and recording of participant’s information. A code number will be used against each name, and personal information would not be published or shared with anyone other than the research team. Data would be entered into a computerized database and would also be imported into a password-protected database that is backed through a secure offsite connection.

The principal investigator (PI) will be responsible for carrying out periodic data checking and would also ensure the systematic patterns, errors, scheduling problems, or overall data integrity. The PI will visit the field sites at least once monthly and monitor the data collection. For the qualitative part, transcribed data will be cross-checked with the field notes. Also, to validate translation (from Bangla to English), the research investigation staff will randomly check the transcribed interviews. No one other than the investigators of the research project will have access to information and data collected from the participants.

Data coding, quality control, and data entry will be done following established procedures at icddr,b. Precoded questionnaire will be used. All data forms will be checked for errors, and necessary corrections will be made before data entry. Quantitative data will be entered using SPSS data entry program (version 20) with built-in range and consistency checks. Frequency distributions will be run to identify outliers. Qualitative data will be entered in MS Word and MS Excel for creating a matrix.

### Statistical Analysis

#### Qualitative Analysis

Qualitative data will be analyzed using a framework approach. After familiarization with the data by listening to tapes, transcribing interviews, reading transcripts, and studying notes to highlight common ideas and recurrent themes, investigators will identify key issues, concepts, and themes by drawing on priority issues and questions raised by the respondent based on the aims of the study. Finally, data will be systematically indexed or coded, synthesized, and interpreted for providing explanations for the findings based on study objectives analyzed under relevant emerging themes and subthemes. Results on the same issues from different types of respondents and areas will be compared to strengthen the validity of the findings.

#### Quantitative Analysis

Descriptive analyses and frequencies of all variables for data visualization will be performed followed by an analytical framework to run analyses. Chi-square tests will be used for categorical variable, and nonparametric *t* test will be used for continuous variables to assess comparisons between different groups. A *P* value <.05 will be considered statistically significant. All statistical analysis will be done using the SPSS software (version 20).

### Ethics Review

This protocol has been approved by the icddr,b Institutional Review Board (IRB). The study protocol was also reviewed by the Director of Compliance of the IRB at the Boston Children’s Hospital in January 2017 with respect to involvement of Boston-based mentors who will not have direct contact with research subjects, will not be involved in subject recruitment or obtaining informed consent, and will not be viewing any data that will have subject identifiers. The study was considered exempt from human subject research review.

All study participants will be recruited following a written informed consent. A trained research staff will explain the nature and purpose of the study, procedures to be followed, potential risks, benefits to be derived, and right to refuse to participate or to withdraw from the study before obtaining consent. All interviews and the sessions would be conducted in a designated private room in one-to-one sessions to ensure participant’s privacy, and no one will be asked any sensitive questions.

Psychosocial counseling will be provided to the mothers free of cost by the designated psychologists deployed at each school. Although the mental health services will target participating mothers, the psychosocial counseling services will be made available to any mother whose child is registered in the schools and who willingly attends the counseling center. However, outcome assessment will be conducted only among the mothers who would be recruited in the intervention phase. All information of the participants will be kept confidential, and no information obtained from the participants will be shared with the school authorities. The interviews would be conducted in a designated private room.

Several mechanisms would be deployed to ensure privacy, anonymity, and confidentiality of data shared by the participants. A unique identification number will be used for tracking each parent and link it to basic addresses for tracking them until data collection and entry are completed. Participant’s names, addresses and other identifiers will only be accessed by the national research staff, coinvestigators, and psychologists; no one other than the national investigators of the research project and members of the Ethical Review Committee of icddr,b will have access to information and data collected from the participants.

### Dissemination

A dissemination seminar will be held locally inviting representatives of the ministry of health, ministry of social welfare, officials, nongovernmental organization (NGO) representatives, relevant professional bodies, researchers, policy makers, program managers, and media for sharing the preliminary findings. To ensure maximum research uptake and further research opportunities, the findings will be also shared with international agencies and donor organizations. Final results will be published in peer-reviewed journals and will be presented in international scientific conferences.

## Results

The trial registration number was obtained in March 2017 (NCT-03025646). Staff training was conducted in January 2017, and the team completed baseline recruitment of 175 mothers by March 2017. We have screened all participants for depression and applied SCID-1 among 65 mothers who had a PHQ-9 score more than 4. We have simultaneously conducted in-depth interviews among 10 mothers and key informant interviews among 8 various stakeholders, including psychiatrists (2), psychologists (2), special educators (2), principals of schools (1), and school managers (1). The remaining surveys will be completed by mid-July 2017. Data entry and data management are ongoing.

Between January and June 2017, the team finalized two intervention modules under the guidance of two different EWGs. A draft Psychosocial Counseling Module (PCM) has been developed by two professional psychologists under the guidance of an EWG (EWG1). Between January and April 2017, the team customized the BPEP training module, and the training module was validated under the guidance of an EWG (EWG2).

In May 2017, counseling centers were set up and psychologists were deployed at the school following training. The school authority helped mothers to fix an appointment if they were interested to meet the psychologists, and the psychologists offered services at the schools between 8:30 AM and 5:00 PM on week days. A total of 41 mothers have attended the counseling centers till the first week of June and participated in two mental health awareness workshops held at each of the schools. Three special educators have been trained in June 2017, and the group initiated training of the participating mothers in July 2017.

## Discussion

### Summary

To our knowledge, this will be the first combined mental health and ASD support intervention for mothers of children with ASD to reduce their burden of depression. The project integrates mental health services and school-based programs targeting ASD. If the proposed intervention strategy is found feasible and effective, the project is likely to significantly improve care for ASD at a low cost in a low-resource country context where the burden of maternal depression is high.

Cost is one of the key barriers to adopting new interventions in the existing programs in low- and middle-income countries. The proposed research will capture the direct cost of the interventions based on actual activities involving real-time tracking of estimate of all possible realistic costs that would be incurred to both the schools (provider cost) and the participating families (out of pocket costs). Due to infeasibility of estimating indirect costs due to productivity losses by parents, only the real-time direct cost estimates are expected to help researchers, institutions, and policy makers to design a large-scale trial for scaling up the intervention in Bangladesh.

### Limitations

The study will only be conducted in urban settings; therefore, we cannot generalize the findings to rural areas of Bangladesh. However, the burden of ASD is likely to be higher in urban areas, and thus, the study will meaningfully contribute to identifying challenges of supporting mental health among a larger group of mothers who have children with ASD. The proposed study only intends to assess the feasibility of the proposed survey; hence, it would not have enough power to estimate the effect of the intervention on maternal depression and performance of children with ASD.

## Appendix

Multimedia Appendix 1 Consent forms.

Multimedia Appendix 2 Study activity flowchart.

## Abbreviations

ADCL: ASD Diagnostic Check-List
ASD: autism spectrum disorder
AWF: Autism Welfare Foundation
BCC: behavioral change communication
BDI: Beck Depression Inventory
BPEP: Bangladesh Parent Empowerment Program
CBT: Cognitive Behavioral Therapy
DALY: disability-adjusted life year
DSM-IV: Diagnostic and Statistical Manual of Mental Disorder, 4th edition
DSMP: Data Safety Monitoring Plan
EQ-5D: Euro-Qol five dimensions questionnaire
EWG: Expert Working Group
icddr,b: International Centre for Diarrheal Disease Research, Bangladesh
IRB: Institutional Review Board
MDD: major depressive disorder
MDE: major depressive episode
NGO: nongovernmental organization
NIMH,B: National Institute of Mental Health, Bangladesh
PHQ-9: Patient Health Questionnaire
PI: principal investigator
SCID: Structured Clinical Interview for DSM-IV
