# Supplementary material for: Implementing a Mental Health Care Program and Home-Based Training for Mothers of Children With Autism Spectrum Disorder in an Urban Population in Bangladesh: Protocol for a Feasibility Assessment Study
Source: JMIR Res Protoc. 2017 Dec 14;6(12):e251. doi: 10.2196/resprot.8260 (PMC5754210; doi:10.2196/resprot.8260)
Supplement: Multimedia Appendix 1 [file resprot_v6i12e251_app1.pdf]

Multimedia Appendix 1: Informed Written Consent form-English

Consent for screening of maternal depression Protocol Title: Feasibility of Implementing a Mental Health Care Program and Home-based Training for Mothers of Children with Autism Spectrum Disorder in an urban population in Bangladesh

PI: Dr Aliya Naheed

Organization: icddr,b

Purpose of the study

Depression is a serious medical illness and an important public health issue. A recent survey by icddr,b, reported that 16% of the adult population suffer from depressive disorder and the rate is higher among female. One research of icddr,b has demonstrated that one out of two mothers of children with ASD (45%) suffer from major depression and may need mental health care supports by a qualified provider. Mothers are primary care givers of children with ASD, and they must have sound mental state for providing care to children at home. Additionally, mothers of child with ASD require special training by qualified providers for enhancing improvement of child performances, and there is scarcity of qualified providers in Bangladesh. Thus it will be important that mothers of children with ASD suffering from depression have access to mental health care and they are also trained for providing better supports for their children care at home.

A group of scientists in icddr,b is going to assess the feasibility of introducing mental health care service in AWF/SWAC in order to provide mental health supports to the mothers of children with ASD and also conduct a home based training for those mothers who suffer from depression in order to strengthen their skills for child care at home. You are a mother of a child with ASD and your child is enrolled in AWF/SWAC. We therefore, would like to invite you to participate in the research and allow us time to describe it to you.

Methods and procedures

If you agree to participate in this research our trained research staffs will obtain a written consent from you and obtain information about you, your child and your family. In this study we would find about a total of 52 to 78 mothers of the children with ASD from two schools (AWF & SWAC) for their recruitment. We will ask you about your socio-demographic information, economic status, information about your child and family, health care seeking pattern, social support system to take care of your child etc. We will conduct preliminary assessment of your depression and assess quality of life. The interview and assessments will take 30 minutes. If we suspect that you may have depression we will further investigate to confirm the diagnosis of depression and it will take additional 30 minutes. If our assessment confirms that you are suffering from depression we will again seek your consent for participating in the intervention and describe the details of the intervention process in a separate session. After 6 months we will again interview you for obtaining some of the information that have already been obtained in the first interview, and assess depression and quality of life, irrespective of being diagnosed as having depression in the first interview.

Risk and benefits

Participation in the study will not involve any major risk. However, after being identified as having depression, a mother may feel stigmatized or frustrated. Counseling or consultation would require time which may not be easy for a mother to manage. Some mother may be diagnosed as having a severe condition and require immediate treatment. Treatment and medication for the mother may incur cost to a mother which would not occur unless a mother is diagnosed as having depression. This interview will take 30 minutes. If any mother assumed having depression primarily then it needs another 30 minutes more to confirm the diagnosis.

In order to reduce the potential risks, each mother with depression would be counseled very closely irrespective of her participation in the intervention. The research team will make appointment at a time and space convenient for the mother in order to collect data. IF any mother needs treatment or hospitalization we will consult with the designated psychiatrists at the collaborating mental health hospital (NIMH,B) and refer the mother to an appropriate facility.

You will not get any financial benefits for participating in this study, but you will know if you have depression at no cost. If you suffer from depression we will invite you to participate in the intervention and you will get psychosocial counseling or treatment as well as training at free of cost. We will refer you to a qualified provider if you suffer from depression or other mental health condition. Your participation in this study will help us to assess depression among mothers of children with ASD in AWF/SWAC and identify those mothers who would be eligible to participate in the intervention and assess if the proposed intervention is feasible for the local setting. The findings of the research will generate essential evidence for developing a suitable strategy for mental health care for mother of children with ASD and advocate the researchers, managers and policy makers for scaling up the intervention at a larger scale in Bangladesh.

Privacy, anonymity and confidentiality

Any information provided by you will be kept with strict confidentiality and no one will have access to your data other than the researchers of this study and the Ethical Review Committee. We will assign a code number to link to your data instead of your name for analyses of data. Your name or other identifiers will not be used in any report and all personal information will be deleted after data analyses.

Future use of information

We will collectively use data provided by you and other mothers in this school for publishing a research report. The report generated from this study might be used in the future to develop appropriate intervention for reduction of depression. The findings of the research will be shared with the policy makers to design appropriate program in order to address depression and strengthen the existing services for ASD in Bangladesh.

Right not to participate and withdraw

Your participation is completely voluntary. You will not be discriminated or harmed by any means if you don’t participate in this study. You will have the right not to answer any question we ask even if you participate. You may also choose to withdraw yourself from the study at any time, even if you agree.

Answering your questions/ Contact persons

For further questions you may call Dr. Aliya Naheed, Principal Investigator of this study over mobile number: 01709651476 or telephone number: 9827075 Extension 2505. If you feel or have any further query about your rights (risk, benefits, other study procedures etc) after joining the study you may also call M A Salam Khan, IRB Secretariate Research Administration at ICDDR, B. The phone contact number of M A Salam Khan is Phone No: 9886498 or PABX 8860523-32 Extension. 3206)

If you agree to participate in our study, please indicate that by putting your signature or your left thumb impression at the specified space below. Thank you very much for your time and cooperation.

Documentation of informed consent & Authorization

- It was given enough time to consider the decision to participate in this research.
- This research has been satisfactorily explained to me, including possible risks and benefits.
- All my questions were satisfactorily answered.
- I understand that participation in this research is voluntary and that I can withdraw at any time.
- I am signing this consent form prior to participation in any research activities.

____________________________ ____________________

Signature of the Participant Date

Research staff's statement & signature

- I have fully explained the research including the possible risks and benefits,
- I have answered and will answer all questions to the best of my ability.
- I have provided a copy of the consent form signed by the respondent

____

____

Signature of the PI or his/her representative Date

Informed Written Consent form-English

(To participate in the intervention phase)

Consent form: Intervention mothers

Protocol Title: Feasibility of Implementing a Mental Health Care Program and Home-based Training for Mothers of Children with Autism Spectrum Disorder in an urban population in Bangladesh

PI: Dr Aliya Naheed

Organization: icddr,b

Purpose of the study:

Depression is a serious medical illness and an important public health issue. A recent survey by icddr,b, reported that 16% of the adult population suffer from depressive disorder and the rate is higher among female. One research of icddr,b has demonstrated that one out of two mothers of children with ASD (45%) suffer from major depression and may need mental health care supports by a qualified provider. Mothers are primary care givers of children with ASD, and they must have sound mental state for providing care to children at home. Additionally, mothers of child with ASD require special training by qualified providers for enhancing improvement of child performances, and there is scarcity of qualified providers in Bangladesh. Thus it will be important that mothers of children with ASD suffering from depression have access to mental health care and they are also trained for providing better supports for their children care at home.

A group of scientists in icddr,b is going to assess the feasibility of introducing mental health care service in AWF/SWAC in order to provide mental health supports to the mothers of children with ASD and also conduct a home based training for those mothers who suffer from depression in order to strengthen their skills for child care at home. You are a mother of a child with ASD and your child is enrolled in AWF/SWAC. We therefore, would like to invite you to participate in the research and allow us time to describe it to you.

Methods and procedures

If you agree to participate in this research our trained research staffs will obtain a written consent from you and invite you for participating in the intervention of mental health service and maternal training program for a period of ~ 6 months. In this study we would find about a total of 52 to 78 mothers of children with ASD from two schools (AWF & SWAC) for their recruitment in the intervention. If you agree we will request you to participate in the following activities.

1. We will conduct an in-depth interview for obtaining your perspectives about introducing a mental health service and training program for mothers at the school setting, barriers of introducing such program and your recommendations for other solutions for reducing depression among mothers of children with ASD. We will also ask you about cost related

to health care services and supports for child care at home, at school and other reasons. The interview may take 30 minutes and we will conduct it at your convenient time.

2. We will refer you to a psychologist who will be deployed at a counseling centre established at your school during school hour and request you to adhere to the counseling session for addressing your depression. The counseling session might take around 1 hour and need minimum 12 sessions. However the number can be more according to the client's requirement.

3. One psychiatrist will visit the centre every month and may invite you for further assessment of your mental health condition and provide treatment, if required. The psychiatrists may need to refer you to a hospital or other facility if your condition demands advance care, if you agree.

4. We will conduct two workshops at two months interval at your school and we will request you to participate in those workshops that will last for no more than one hour per session.

5. We will organize a group session by a special educator to train you how to take better care of your child at home and it will take about 2 weeks time to complete training following a pre designed module. Overall there would be around 6 sessions each session will be around 2 hours.

6. A special educator will visit you at home to provide refresher training up to 4 months following the initial group training conducted at the school. During the monthly home visits, the educator will ask you about costs incurred by you or your family for obtain any service related to health care and training either for you or your child. The educator will also assess performance of your child using a assessment form (ADCL) and would refer him/her to a clinical expert for any additional need either identified by you or the educator herself.

7. Upon completion of the 6 months intervention, we will interview you to assess your perspectives about the services offered to you through the school setting, assess the status of your depression and performance of child in order to assess impact of the intervention.

Risk and benefits

Participation in the intervention will not involve any major risk. However, after being identified as having depression, a mother may feel stigmatized or frustrated. The counseling session might take around 1 hour depending on her condition and about 2 hours to attend the training for 6 days, which may not be easy for a mother to manage.. Some mothers may be diagnosed as having a severe condition and require immediate treatment. Treatment and medication for the mother may incur cost to a mother which would not occur unless a mother is diagnosed as

having depression. In order to reduce the potential risks, each mother with depression would be counseled very closely irrespective of her participation in the intervention. The research team will make appointment at a time and space convenient for the mother in order to collect data. If any mother needs treatment or hospitalization we will consult with the designated psychiatrists at the collaborating mental health hospital (NIMH,B) and refer the mother to an appropriate facility. All costs of treatment, medication and hospitalization at the NIMH,B would be borne by the hospital. Other cost related to treatment will not be borne by the study, but we will facilitate so that mothers can get the best standard of treatment available in the country.

Privacy, anonymity and confidentiality

Any information provided by you will be kept with strict confidentiality and no one will have access to your data other than the researchers, psychologists, psychiatrics involving of this study and the Ethical Review Committee. During psychological intervention a record sheet containing name, address and personal information will be maintained to track the participants for providing multiple therapeutic sessions. The Principal Investigator and her team will keep confidentiality and maintain a record sheet obtaining from intervened mothers of the study as well as assure the study data by securing them in a locked cabinet. We will assign a code number to link to your data instead of your name for analyses of data. Your name or other identifiers will not be used in any report and all personal information will be deleted after data analyses.

Future use of information

We will collectively use data provided by you and other mothers in this school for publishing a research report. The report generated from this study might be used in the future to develop appropriate intervention for reduction of depression. The findings of the research will be shared with the policy makers to design appropriate program in order to address depression and strengthen the existing services for ASD in Bangladesh.

Right not to participate and withdraw

Your participation is completely voluntary. You will not be discriminated or harmed by any means if you don't participate in this study. You will have the right not to answer any question we ask even if you participate. You may also choose to withdraw yourself from the study at any time, even if you agree.

Answering your questions/ Contact persons

For further questions you may call Dr. Aliya Naheed, Principal Investigator of this study over mobile number: 01709651476 or telephone number: 9827075 Extension 2505. If you feel or have any further query about your rights (risk, benefits, other study procedures etc) after joining the study you may also call M A Salam Khan, IRB Secretariate Research Administration at

ICDDR, B. The phone contact number of M A Salam Khan is Phone No: 9886498 or PABX 8860523-32 Extension. 3206)

If you agree to participate in our study, please indicate that by putting your signature or your left thumb impression at the specified space below. Thank you very much for your time and cooperation.

Documentation of informed consent & Authorization

- It was given enough time to consider the decision to participate in this research.
- This research has been satisfactorily explained to me, including possible risks and benefits.
- All my questions were satisfactorily answered.
- I understand that participation in this research is voluntary and that I can withdraw at any time.
- I am signing this consent form prior to participation in any research activities.

____

____

Signature of the Participant Date

Research staff's statement & signature

- I have fully explained the research including the possible risks and benefits,
- I have answered and will answer all questions to the best of my ability.
- I have provided a copy of the consent form signed by the respondent.

____

____

Signature of the PI or his/her representative Date

Consent form: For KII with mangers, providers and policy makers

Protocol No: 16070

Protocol Title: Feasibility Study of Implementing a Mental Health Care Program and Home-based Training for Mothers of Children with Autism Spectrum Disorders.

PI: Dr Aliya Naheed

Organization: icddr,b

Purpose of the research

Background

Depression is a serious medical illness and an important public health issue. A recent survey by icddr,b, reported that 16% of the adult population suffer from depressive disorder and the rate is higher among female. One research of icddr,b has demonstrated that one out of two mothers of children with ASD (45%) suffer from major depression and may need mental health care supports by a qualified provider. Mothers are primary care givers of children with ASD, and they must have sound mental state for providing care to children at home. Additionally, children with ASD require special training by qualified providers for enhancing improvement of child performances, and there is scarcity of qualified providers in Bangladesh. Thus it will be important that mothers of children with ASD suffering from depression have access to mental health care and they are also trained for providing better supports for their children care at home.

A group of scientists in icddr,b is going to assess the feasibility of introducing mental health care service in AWF/SWAC in order to provide mental health supports to the mothers of children with ASD and also conduct a home based training for those mothers who suffer from depression in order to strengthen their skills for child care at home.

Since you have been contributing as a manger/ provider/ policy maker for providing care for children with ASD we would like to conduct an in-depth interview with you in order to learn about your experiences and hear your perspective about the barriers and opportunities of the proposed intervention as well as your recommendations for future considerations. We therefore, would like to invite you to participate in this study and allow us time to describe it to you.

Methods and procedures:

If you agree, we will obtain a written consent from you and learn about your experiences about with ASD as a manger/ provider/ policy maker. We will set an appointment with you at your convenience and use an interview guideline to for obtaining your perspective about the barriers and opportunities of the proposed intervention at school setting. We will also capture your

perception about the existing mental health services and training for parents in the local contexts and potential of the intervention for reducing depression among mothers of children with ASD. Finally we will try to get your recommendations how the proposed intervention can be scalable for future considerations.

Risk and benefits

Participation in this study involves no risk and we will conduct the interview at a time convenient for you. We will not ask you any sensitive question that may offend you. You will not get any financial benefits for participating in this study, but your participation in this study will help us to assess if the proposed intervention would be feasible for the local setting. The findings of the research will generate essential evidence for developing a suitable strategy for mental health care for mother of children with ASD and advocate the researchers, managers and policy makers for designing appropriate program to scaling up the intervention at a larger scale in Bangladesh. Additionally, the findings of the interview with you and other relevant stakeholders including mangers and providers will direct the policy makers to address this problem and reinforce the existing mental health services in autism.

Privacy, anonymity and confidentiality

Any information provided by you will be kept with strict confidentiality and no one will have access to your data other than the researchers of this study and the Ethical Review Committee. We will assign a code number to link to your data instead of your name for analyses of data. Your name or other identifiers will not be used in any report and all personal information will be deleted after data analyses.

Future use of information

We will collectively use data provided by you and other mothers in this school for publishing a research report. The report generated from this study might be used in the future to develop appropriate intervention for reduction of depression. The findings of the research will be shared with the policy makers to design appropriate program in order to address depression and strengthen the existing services for ASD in Bangladesh.

Right not to participate and withdraw

Your participation is completely voluntary and will not be penalized if you may choose not to participate in this study. You will have the right not to answer any question we ask even if you participate. You may also choose to withdraw yourself from the study at any time, even if you agree.

Answering your questions/ Contact persons

For further questions you may call Dr. Aliya Naheed, Principal Investigator of this study over mobile number: 01709651476 or telephone number: 9827075 Extension 2505. If you feel or have any further query about your rights (risk, benefits, other study procedures etc) after joining the study you may also call M A Salam Khan, IRB Secretariate Research Administration at ICDDR, B. The phone contact number of M A Salam Khan is Phone No: 9886498 or PABX 8860523-32 Extension. 3206)

If you agree to participate in our study, please indicate that by putting your signature or your left thumb impression at the specified space below. Thank you very much for your time and cooperation.

____

____

Signature or left thumb impression of the participant Date

____

____

Signature of the PI or his/her representative Date
