# Supplementary figures and images for: Implementing a Mental Health Care Program and Home-Based Training for Mothers of Children With Autism Spectrum Disorder in an Urban Population in Bangladesh: Protocol for a Feasibility Assessment Study
Source: JMIR Res Protoc. 2017 Dec 14;6(12):e251. doi: 10.2196/resprot.8260 (PMC5754210; doi:10.2196/resprot.8260)

## Multimedia Appendix 2: Study activity flow chart

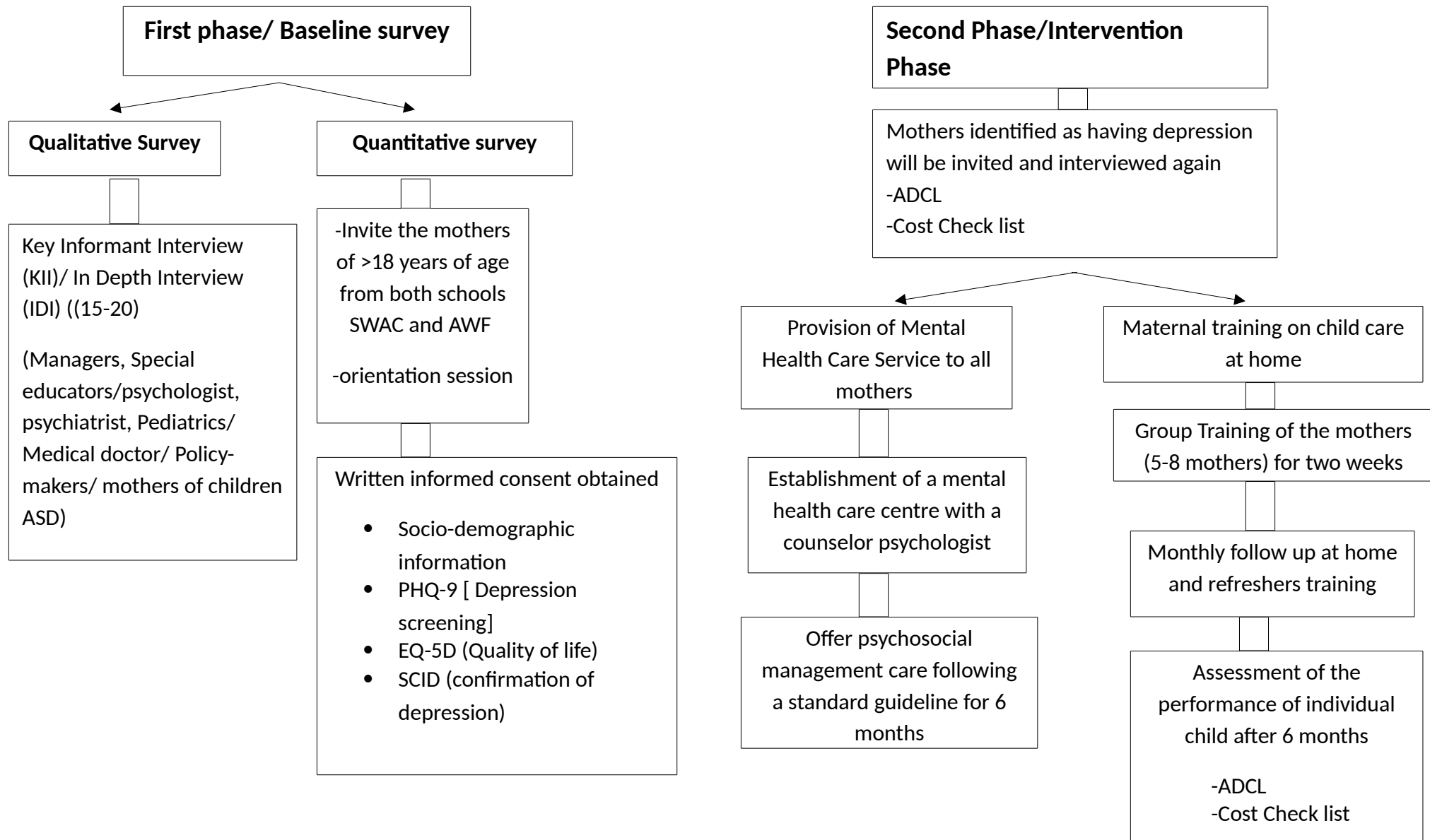

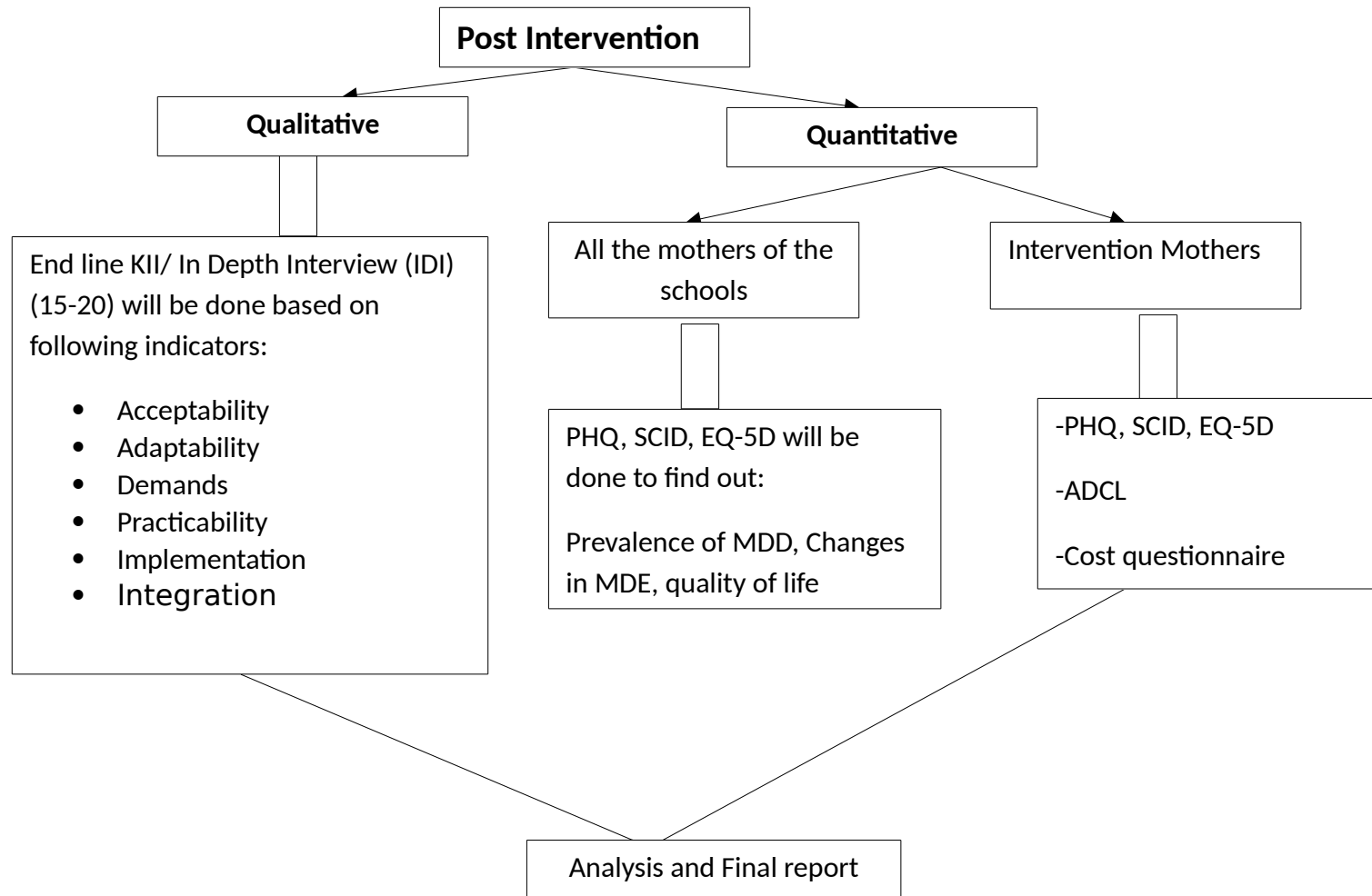

Supplement: Multimedia Appendix 2 [file resprot_v6i12e251_app2.pdf]
